# Sexual Function in Women with Stress Urinary Incontinence Treated with the SPARC Sling System

**DOI:** 10.1155/2013/957547

**Published:** 2013-07-14

**Authors:** Badereddin Mohamad Al-Ali, Rany Shamloul, Georg C. Hutterer, Erika Puchwein, Karl Pummer, Alexander Avian, Günter Primus

**Affiliations:** ^1^Department of Urology, Medical University of Graz (MUG), Auenbruggerplatz 7, 8036 Graz, Austria; ^2^Department of Urology, University of Ottawa, Canada; ^3^Department of Statistics, Medical University of Graz (MUG), Auenbruggerplatz 2/9/V, 8036 Graz, Austria

## Abstract

*Aim*. To evaluate the impact of SPARC on female sexual function. *Methods*. 151 women with a mean age of 60 ± 11.90 and SUI had a complete urodynamic investigation and underwent SPARC operation. 98 women completed the validated female sexual function index questionnaire (FSFI) at baseline and 94 women at follow-up. A minimum follow-up of 12 months was required for study inclusion. *Results*. 52/98 women were sexually active at baseline. Postoperatively only 33 patients were sexually active. The FSFI score of all 33 pre- and postoperative sexually active women increased from 25.3 ± 5.7 at baseline to 27.4 ± 4.8 at follow-up (*P* = 0.1). Scores of women with reduced sexual function at baseline increased significantly in the domains desire, arousal, and lubrication as well as orgasm and satisfaction and total FSFI-score (*P* = 0.002) postoperatively. *Conclusions*. Our results suggest that the SPARC-sling procedure for SUI did not negatively interfere with female sexual function.

## 1. Introduction

Female urinary incontinence is defined by the International Continence Society as the complaint of any involuntary leakage of urine [1]. It is a common condition with wide range prevalence between 12.8% and 46.0% [2]. Stress urinary incontinence (SUI) is the involuntary leakage of urine on effort or exertion [1]. Urinary leakage can have a dramatic effect on the quality of female sexual life and may lead to complete abandon of sexual activity in a high proportion of cases [2–7]. Moreover, middle-aged women, who are sexually active, are likely to suffer from SUI [8, 9]. Indeed, it was confirmed that SUI could have dramatic impacts on the sexual function in middle-aged sexually active women; proposed mechanisms include dyspareunia and coital incontinence [10–15].

Surgical treatment of SUI in women offers relatively high success rates and immediate improvement of SUI symptoms. The Female Stress Urinary Incontinence Clinical Guideline Panel of the American Urological Association found that pubovaginal slings, midurethral tapes, and retropubic suspensions were the most effective surgical techniques for SUI in women [16]. 

Several long-term outcome data have documented favorable efficacy and safety effects of the tension-free vaginal tape (TVT) technique [17–19] and the suprapubic arch (SPARC; American Medical Systems, Minnetonka, MN, USA) placement [20–23]. The SPARC sling system, approved by the US Food and Drug Administration in 2001, represents a modification of the TVT, which is the most popular and widely used sling system [24]. Both sling materials consist of a loosely woven monofilament polypropylene mesh. The major difference between SPARC and TVT is trocar size and route of delivery. The aim of this study was to evaluate the effects of the SPARC sling system on the female sexual function. We performed only SPARC operation for SUI because we believe that retropubic midurethral slings have better results in comparison to transobturator slings and we do not have experience with transobturator slings.

## 2. Patients and Methods

The Ethics Committee of the Medical University of Graz, Austria approved this retrospective study. 

The FSFI was developed as a brief, multidimensional self-report instrument for assessing key dimensions of sexual function in women. The scale consists of 19 items that assess sexual function over the past 4 weeks and yield domain scores in six areas: sexual desire, arousal, lubrication, orgasm, satisfaction, and pain. The measure was validated on an initial sample of women with female sexual arousal disorder (FSAD) and a control sample of women without sexual difficulties [25]. Based on sensitivity and specificity analyses and the CART procedure, an FSFI total score of 26.55 was found to be the optimal cutoff score for differentiating women without sexual dysfunction from those with sexual dysfunction. 

The individual domain scores and full-scale score of the FSFI can be derived from the computational formula. For individual domain scores, add the scores of the individual items that comprise the domain and multiply the sum by the domain factor; add the six domain scores to obtain the full-scale score.

 Women were eligible for the SPARC surgery if they had predominant SUI symptoms, a positive cough stress test, and a bladder capacity >200 mL. Patients with previous failed incontinence surgery, mixed urinary incontinence (MUI), or previous gynecological surgery were included. Women with neurological findings or vaginal support defects greater than second stage according to the Pelvic Organ Prolapse Quantification system were excluded. Objective cure was defined as pad weight 0-1 g and a negative cough test in a standing position. Subjective cure was defined as no use of pads according to the micturition diary. Improvement was defined as urine loss only during cough test, a pad weight of >1–5 g, and overall patient satisfaction (by responding “Yes” to the question. Are you satisfied with the degree of urinary continence achieved after the operation?) A written informed consent was obtained from all patients. A minimum follow-up of 12 months was required for study inclusion. Concomitant gynecologic surgery or prolapse repair were not performed in our patients; we performed only SPARC operation in our patients without concomitant gynecologic surgery. We performed SPARC only after hysterectomy.

Statistical analyses were performed using Statistical Package for Social Sciences, version 11.5 (SPSS, Chicago, IL, USA). The mean values were analyzed using the nonparametric Wilcoxon signed rank test. *P* ≤ 0.05  was considered statistically significant.

## 3. Results

Between June 2001 and March 2009, 151 women with SUI and a mean age of 60 ± 11.9 years underwent SPARC sling placement at our institution. Of these, 98 (64.9%) women completed the validated FSFI questionnaire at baseline and 94 (62.3%) women at follow-up. Mean follow-up was 4.71 ± 2.42 years. 52/98 (53.1%) women with a mean age of 58.7 ± 10.4 years were sexually active at baseline and 46/98 (46.9%) women with a mean age of 69 ± 10.9 were sexually inactive at baseline. Preoperatively sexual activity was normal (FSFI > 26.55) in 24/52 (46.2%) with a mean total FSFI score of 30 ± 2.1, and 28/52 (53.8%) showed a reduced sexual function (FSFI < 26.55) with a mean total FSFI score of 20.3 ± 5.4. Patient's characteristics are shown in Table 1. The reasons for sexual inactivity prior surgery are shown in Table 2.

33/52 (63.5%) sexual active women who had the same conditions concerning relationship and personal circumstances pre- and postoperatively were eligible for analysis at follow-up. 19/52 (36.5%) preoperatively sexual active women were excluded due to various reasons. 13/19 (68.4%) became sexually inactive (Table 3) and 4/19 women had postoperatively no partner and they masturbate. In these four women the total FSFI score decreased from 24.8 ± 6.6 to 11.9 ± 4.6 (*P* = 0.07) as well as all subdomains (*P* = 0.07) with the exception of orgasm (*P* = 0.3), showing a clear trend of deterioration of their total sexual function.

41/46 (89.1%) of the sexually inactive women prior surgery were still sexually inactive at follow-up, 3/46 women became sexual active after being cured from SUI, and 2 women presented preoperative data only.

Analysis of the FSFI questionnaire subdomain desire (questions 1 and 2) at baseline showed a score of 3.4 ± 0.9 for sexual active women and 1.5 ± 0.7 for sexual inactive women (*P* = 0.000) and 3.1 ± 1.3 and 1.5 ± 0.8 (*P* = 0.000) at follow-up, respectively.

At baseline 15/33, (45.5%) women had normal sexual function and 18/33 (54.5%) women had a reduced sexual function. At follow-up 18/33 women showed normal sexual function (54.5%) and 15/33 (45.5%) women had reduced sexual function. 

Three women with reduced sexual function prior surgery achieved normal sexual function postoperatively.

Total FSFI-score remained unchanged (*P* = 0.1) in all 33 pre- and postoperative sexual active women, as the subscores did of the various domains, with the exception of the domains desire (*P* = 0.05) and satisfaction (*P* = 0.04) (Table 4). Scores of women with reduced sexual function at baseline increased significantly in the domains desire, arousal, lubrication, orgasm, satisfaction, and total FSFI-score postoperatively, but sexual function remained unchanged in women with normal sexual function. Results of the different subgroups are shown in Table 5.

According to satisfaction 16/33, (48.5%) women showed an increase in their scores, 12 remained unchanged, and five showed a decrease in their scores. More than 50% of all pre- and postoperative sexual active women showed a higher score postoperatively (Table 6). 12 women with a reduced sexual function at baseline had postoperatively a higher FSFI score (Table 7).  Table 8 shows change in scores for the different questions from before surgery to after surgery in all pre- and postoperative women with normal sexual function.

Concerning complications, one patient showed asymptomatic vaginal sling erosion (approximately 1 mm) 4 years after surgery. Due to the small size of the lesion and the total absence of symptoms, no treatment was necessary at this stage, but annual gynaecological examination was recommended. Urinary retention was observed in one patient and significant postvoid residual (>200 mL) in three patients. These complications were successfully treated by immediate loosening of the sling. Perforation of the bladder occurred in four patients with no need of open revision.

De novo urgency symptoms developed in four patients and they improved on anticholinergic medication.

## 4. Discussion

Although male sexual dysfunction received more investigations and attention, female sexual dysfunction remains largely underinvestigated. This fact led us to perform our study.

In this study, we report the impact of the SPARC sling system on the female sexual function in women suffering from SUI. Women undergoing the SPARC sling operation experienced no deterioration of their FSFI scores. Our results do not show any deterioration of sexual function after surgery in contrary to previously reported data that sling operations may cause increased dyspareunia [26, 27]. Proposed mechanisms that may lead to these unfavorable sexual function outcomes include failure of the sling operation to improve the SUI leading to persistence of the same preoperative sexual dysfunctions with fear of coital incontinence that leads to decreased sexual desire. Moreover, adverse effects may occur despite the cure of the SUI as the sling itself may result in interference with vaginal innervations leading to decreased genital sensation, decreased vaginal lubrication, and vaginal erosion [28]. 

Goldstein et al. [28] found a significant worsening only in the domain score of orgasmic function, suggesting that TVT can adversely affect the women's ability to reach orgasm. A vascular or neuronal damage to the anterior vaginal wall or clitoris erectile tissue during midurethral sling placement may interfere with normal response to sexual stimulation. Our results suggest that the likelihood of a deterioration of the sexual function after SPARC procedure is minimal in accordance with previously reports with other slings supporting the hypothesis that incontinence surgery may improve sexual relations even when it does not directly influence symptoms arising during intercourse like dyspareunia and loss of libido.

Additionally, a recent study showed that midurethral slings could interfere with clitoral blood flow and subsequently clitoral erection [29]. In contrary to these reports, other robust studies and excellent reviews showed that TVT slings might actually improve sexual function. In an excellent review, Serati et al. [10] analyzed 14 different studies on female sexual function after various sling operations Tension-Free Vaginal Tape (TVT), tension free vaginal tape-obturator (TVT-O), transobturator tape (TOT) and reported that sexually active women, who underwent sling procedures for SUI, may not experience any deterioration but even experience some improvement of their sexual function, with a potential risk of developing dyspareunia (<15%). Moreover, other specific studies demonstrated that TVT slings might significantly improve the sexual lives of women with SUI [30, 31].

Pace et al. [30] showed in their prospective study that 90.1% of sexually active women reported a significant improvement in their sexual life, while only 9.9% complained a decline in their sexual quality of life but not as a consequence of the surgical procedure. Women who had a worse sexual activity after the procedure (TVT or TOT) were satisfied with the operation for the correction of SUI, but they reported loss of libido and vaginal itching as the main reason for the decline of sexual function. They found an improvement of the FSFI score in both TVT and TOT procedure without worsening of sexual function or sexual activity after the surgical treatment and they concluded patients could be reassured that these operations will not affect their sexual life. 

The SPAR-sling procedure was primarily invented to decrease organ injury, of the bowel, and lower urinary tract that sometimes occur during implantation of the TVT trocar [32], which also represents a significant risk of injury to the pelvic vasculature [21]. 

Kuhn et al. [33] reported that sexual function in patients with de novo dyspareunia is likely to improve after sling removal but not in all domains; considering the route of sling insertion, there is some evidence that transobturator tapes cause more dyspareunia than classical retropubic tapes (SPARC); this is in agreement with our results.

Unfortunately randomized trials with sufficient power to determine which route is best regarding sexual function are missing.

Of the 52 preoperative sexual active women in our study only 33 patients remained sexually active and showing the same conditions pre- and postoperatively. 13/52 women were postoperative abstinent from any sexual activity for a variety of reasons but not tape related. 4 women are divorced postoperative and perform their sexual activity by masturbation and in 2 only preoperative data are present.

The majority of studies which focused on the impact of TVT procedure on the sexual function of women suggest that sexual function is not changed by the procedure, which is in agreement with our results.

Improvement in sexual function may be related to an improvement in self-esteem, which would affect the partners' relationship. Sexual function is a very complex issue and a lot of different factors may influence women's sexuality including hormonal changes, medications, social situation, relationship, availability of a partner, and own health status and of the partner.

Certainly, more in depth studies examining vaginal structural changes with adequate assessment of the vaginal blood flow are needed to understand the favorable changes the SPARC sling has on the sexual function. Finally, our study is limited by the lack of a control group, the low number of patients involved, and the absence of multiple postoperative follow-up sessions. 

## 5. Conclusions

These results suggest that the SPARC-sling procedure for SUI does not worsen sexual function in women. In fact, all domains of the FSFI improved after-SPARC implantation. Additional prospective studies are warranted to verify these preliminary findings and compare the impact of SPARC with that of other anti-incontinence procedures.

## Figures and Tables

**Table 1:** Preoperative characteristic of sexual active and inactive women.

	Sexual active (*N* = 52)	Sexual inactive (*N* = 46)	*P* value
Age (years)*	58.7 ± 10.4 (34–76)	69 ± 10.9 (40–89)	0.000
Parity*	2.1 ± 1.1 (0–4)	2.4 ± 1.4 (0–6)	0.3
Body mass index*	27.6 ± 4.8 (17.9–40.7)	28.4 ± 4.8 (19.8–42)	0.3
Menopause (%)	34/52 (65.4)	42/46 (91.3)	0.003
Premenopausal (%)	18/52 (34.6)	4/46 (8.7)	0.003
Hysterectomy (%)	16/52 (30.8)	22/46 (47.8)	0.01
Hormone replacement (%)	9/52 (17.3)	4/46 (8.7)	0.3
Antidepressant (%)	11/52 (21.2)	15/46 (32.6)	0.3
Diabetes (%)	3/52 (5.8)	6/46 (13)	0.3
Smokers (%)	3/52 (5.8)	2/46 (4.3)	1
Marital status			
Married (%)	35/52 (67.3)	12/46 (26.1)	0.000
Divorced (%)	13/52 (25.0)	8/46 (17.4)	0.5
Widowed (%)	4/52 (7.7)	26/46 (56.5)	0.000

*Mean, standard deviation, and range.

**Table 2:** Causes of sexual inactivity at baseline.

Cause	*N* = 46	%
Lack of partner	31/46	67.4
Erectile dysfunction of partner after RRPE	3/46	6.5
Due to SUI	3/46	6.5
Illness of partner	2/46	4.3
Neurologic due to spinalstenosis	1/46	2.2
Raped	1/46	2.2
Death of son	1/46	2.2
Unknown	4/46	8.7

RRPE: radical retropubic prostatectomy.

SUI: stress urinary incontinence.

**Table 3:** Causes of sexual inactivity after surgery in sexual active women at baseline.

Cause	*N*	%
Lack of partner	6	46.1
Erectile dysfunction of partner after RRPE	1	7.7
Illness of patient	3	23.1
Illness of partner	2	15.4
Rape	1	7.7

Total	13	100

RRPE: retropubic radical prostatectomy.

**Table 4:** FSFI domains' score of all pre- and postoperative sexual active women before and after surgery.

Domains	Baseline	After SPARC	*P* value
Desire	3.4 ± 0.9 (1.2–4.8)	3.7 ± 0.8 (2.4–6.0)	0.05
Arousal	3.9 ± 1.1 (0.9–6.0)	4.2 ± 0.9 (2.4–5.4)	0.2
Lubrication	4.5 ± 1.4 (1.2–6.0)	4.7 ± 1.2 (1.2–6.0)	0.4
Orgasm	4.1 ± 1.3 (1.2–6.0)	4.4 ± 1.3 (1.2–6.0)	0.4
Satisfaction	4.5 ± 1.3 (1.2–6.0)	4.9 ± 1.2 (1.2–6.0)	0.04
Pain	4.9 ± 1.3 (1.6–6.0)	5.3 ± 0.9 (2.8–6.0)	0.1
FSFI total score	25.3 ± 5.7 (10.2–34.8)	27.4 ± 4.8 (18.1–35.6)	0.1
Number of patients	33	33	

FSFI: female sexual function index.

Domain values are presented as mean, standard deviation, and range.

**Table 5:** FSFI domains' score of all pre- and postoperative sexual active women with normal and reduced sexual function before and after surgery.

Domains	NSF preop.	NSF postop.	*P* value	RSF preop.	RSF postop.	*P* value
Desire	3.7 ± 0.8 (2.4–4.8)	3.7 ± 0.7 (2.4–4.8)	0.9	3.1 ± 0.9 (1.2–4.2)	3.7 ± 0.9 (2.4–6)	0.03
Arousal	4.7 ± 0.6 (3.9–6)	4.6 ± 0.6 (3.3–5.4)	0.6	3.2 ± 1 (0.9–4.5)	3.9 ± 1 (2.4–5.4)	0.01
Lubrication	5.5 ± 0.6 (4–6)	5.4 ± 0.6 (3.6–6)	0.6	3.7 ± 1.3 (1.2–6)	4.2 ± 1.4 (1–6)	0.04
Orgasm	5.2 ± 0.7 (4–6)	4.8 ± 1.3 (1.2–6)	0.09	3.2 ± 1 (1.2–4.4)	4.1 ± 1.2 (1.2–5.6)	0.02
Satisfaction	5.3 ± 1.2 (1.2–6)	5.2 ± 1.4 (1.2–6)	0.9	3.8 ± 1 (1–5)	4.7 ± 1 (2–6)	0.01
Pain	5.8 ± 0.5 (4–6)	5.7 ± 0.5 (5-6)	0.9	4.2 ± 1.3 (1.6–6)	5 ± 1.1 (3–6)	0.1
FSFI total score	30.2 ± 2.1 (26.9–34.8)	29.6 ± 3.3 (24.2–35.6)	0.8	21.3 ± 4.3 (10.2–26.1)	25.5 ± 5.1 (18.1–35)	0.002
Number of patients	15	15		18	18	

NSF: women with normal sexual function (FSFI > 26.55).

RSF: women with reduced sexual function (FSFI < 26.55).

FSFI: female sexual function index.

Domain values are presented as mean, standard deviation, and range.

**Table 6:** Change in scores for the different questions from before surgery to after surgery in all pre- and postoperative sexual active women.

Variable	Higher		Unchanged		Lower		Total	
	*N*	%	*N*	%	*N*	%	*N*	%
Desire	12	36.4	15	45.4	6	18.2	33	100
Arousal	12	36.4	14	42.4	7	21.2	33	100
Lubrication	10	30.3	14	42.4	9	27.3	33	100
Orgasm	11	33.3	13	39.4	9	27.3	33	100
Satisfaction	16	48.5	12	36.4	5	15.1	33	100
Pain	10	30.3	18	54.6	5	15.1	33	100
Total FSFI	18	54.5	6	18.2	9	27.3	33	100

**Table 7:** Change in scores for the different questions from before surgery to after surgery in all pre- and postoperative women with reduced sexual function (FSFI < 26.55).

Variable	Higher		Unchanged		Lower		Total	
	*N*	%	*N*	%	*N*	%	*N*	%
Desire	9	50.0	6	33.3	3	16.7	18	100
Arousal	8	44.4	6	33.3	4	22.2	18	100
Lubrication	7	38.9	6	33.3	5	27.8	18	100
Orgasm	9	50.0	5	27.8	4	22.2	18	100
Satisfaction	12	66.6	3	16.7	3	16.7	18	100
Pain	9	50.0	7	38.9	2	11.1	18	100
Total FSFI	12	66.7	2	11.1	4	22.2	18	100

**Table 8:** Change in scores for the different questions from before surgery to after surgery in all pre- and postoperative women with normal sexual function (FSFI > 26.55).

Variable	Higher		Unchanged		Lower		Total	
	*N*	%	*N*	%	*N*	%	*N*	%
Desire	3	20.0	9	60.0	3	20.0	15	100
Arousal	4	26.7	8	53.3	3	20.0	15	100
Lubrication	3	20.0	8	53.3	4	26.7	15	100
Orgasm	2	13.3	8	53.3	5	33.3	15	100
Satisfaction	4	26.7	9	60.0	2	13.3	15	100
Pain	1	6.7	11	73.3	3	20.0	15	100
Total FSFI	6	40.0	4	26.7	5	33.3	15	100
